# Scale-up and process integration of sugar production by acidolysis of municipal solid waste/corn stover blends in ionic liquids

**DOI:** 10.1186/s13068-016-0694-8

**Published:** 2017-01-05

**Authors:** Chenlin Li, Ling Liang, Ning Sun, Vicki S. Thompson, Feng Xu, Akash Narani, Qian He, Deepti Tanjore, Todd R. Pray, Blake A. Simmons, Seema Singh

**Affiliations:** 1 Advanced Biofuels (and BioProducts) Process Demonstration Unit, Lawrence Berkeley National Laboratory, 1 Cyclotron Road, Berkeley, CA 94720 USA; 2 Energy and Environmental Science and Technology, Idaho National Laboratory, 2525 North Fremont Ave, Idaho Falls, ID 83415 USA; 3 Biological Systems and Engineering Division, Lawrence Berkeley National Laboratory, 1 Cyclotron Road, Berkeley, CA 94720 USA; 4 Joint BioEnergy Institute, Lawrence Berkeley National Laboratory, 1 Cyclotron Road, Berkeley, CA 94720 USA; 5 Biomass Science and Conversion Technology Department, Sandia National Laboratories, 7011 East Avenue, Livermore, CA 94551 USA

**Keywords:** Scale-up, Ionic liquid, Acidolysis, MSW/CS blends, Reactor compatibility

## Abstract

**Background:**

Lignocellulosic biorefineries have tonnage and throughput requirements that must be met year round and there is no single feedstock available in any given region that is capable of meeting the price and availability demands of the biorefineries scheduled for deployment. Significant attention has been historically given to agriculturally derived feedstocks; however, a diverse range of wastes, including municipal solid wastes (MSW), also have the potential to serve as feedstocks for the production of advanced biofuels and have not been extensively studied. In addition, ionic liquid (IL) pretreatment with certain ILs is receiving great interest as a potential process that enables fractionation of a wide range of feedstocks. Acid catalysts have been used previously to hydrolyze polysaccharides into fermentable sugars following IL pretreatment, which could potentially provide a means of liberating fermentable sugars from lignocellulose without the use of costly enzymes. However, successful optimization and scale-up of the one-pot acid-assisted IL deconstruction for further commercialization involve challenges such as reactor compatibility, mixing at high solid loading, sugar recovery, and IL recycling, which have not been effectively resolved during the development stages at bench scale.

**Results:**

Here, we present the successful scale-up demonstration of the acid-assisted IL deconstruction on feedstock blends of municipal solid wastes and agricultural residues (corn stover) by 30-fold, relative to the bench scale (6 vs 0.2 L), at 10% solid loading. By integrating IL pretreatment and acid hydrolysis with subsequent centrifugation and extraction, the sugar and lignin products can be further recovered efficiently. This scale-up development at Advanced Biofuels/Bioproducts Process Demonstration Unit (ABPDU) will leverage the opportunity and synergistic efforts toward developing a cost-effective IL-based deconstruction technology by drastically eliminating enzyme, reducing water usage, and simplifying the downstream sugar/lignin recovery and IL recycling.

**Conclusion:**

Results indicate that MSW blends are viable and valuable resource to consider when assessing biomass availability and affordability for lignocellulosic biorefineries. This scale-up evaluation demonstrates that the acid-assisted IL deconstruction technology can be effectively scaled up to larger operations and the current study established the baseline of scaling parameters for this process.

## Background

Renewable biomass represents an abundant source of carbon neutral domestic energy, and its use for biofuels is attracting considerable attention in the US and worldwide as a strategy to mitigate climate change, secure a constant energy supply, and improve rural economies [1]. The success of biofuel and biochemical industries depends on a reliable supply of high-quality biomass, available at a cost that enables meeting the cellulosic biofuel and business profitability targets [2–5]. Some challenges include securing cost-competitive reliable sources of feedstocks in quantities large enough to meet our energy needs; carrying capacity of infrastructures to harvest/collect, sort, and pre-process biomass feedstocks and transport and store products; technologies capable of converting these into consumable cost-competitive energy products; and ensuring environmental and public health protection and benefits. Many research and development efforts, however, have been historically focused on the utilization of agriculturally derived cellulosic feedstocks, such as agricultural residues, perennial grasses, woody perennials, and forest products. A diverse range of wastes, including municipal solid wastes (MSW), also possess great potentials to serve as feedstocks for the production of biofuels and biochemicals [6–10], and have not been extensively studied to date in terms of conversion efficiency and scale-up performance.

Compared with the seasonal availability of agricultural wastes, MSW has the advantages of year-round availability, an established collection infrastructure and potential availability at negative cost [11]. An efficient use of MSW would not only benefit the biofuel industry, but also reduce landfill disposal [7]. Recent reports projected that an estimated 44.5 million dry tons of MSW will be available in 2022 in the United States, among which mixed paper waste is one of the major components, representing about 30% of total MSW [12]. In addition, biomass feedstock costs remain a large contributor to biofuel production costs and US DOE has a cost target of $80/dry metric ton at year 2017 [6]. While improvements in technology for biomass harvest and collection, storage, preprocessing, and handling and transportation will help to meet this goal, reductions in grower payment will be required since it is one of the largest contributors to costs. One promising alternative to reduce the cost is to blend more expensive high-quality feedstocks with lower cost, lower quality feedstocks such as MSW so that the overall quality still meets the required specifications for the biorefinery [6, 13]. Given the seasonal availability of plant-derived feedstocks, and the continual supply and established infrastructure for MSW, it will be advantageous and important to consider use of MSW as an advanced biofuels’ feedstock, especially as a blending agent to help normalize the composition of the biomass inputs to a biorefinery which has a narrow tolerance to variation in biomass composition [13].

Another key factor in the successful large-scale production of cellulosic biofuels is efficient and cost-effective biomass deconstruction process for fermentable sugar conversion. Among the various leading pretreatment technologies, certain imidazolium-based ionic liquids (ILs) have been shown to efficiently fractionate biomass and provide fermentable sugars for downstream production of advanced biofuels [14, 15]. Previous work illustrated several favorable properties of IL pretreatment for biomass deconstruction at bench scale, including biomass dissolution and cell wall disruption, reduced cellulose crystallinity, reduced lignin content, and feedstock agnostic [16, 17]. Studies have been published by our group on the scale-up demonstration of IL pretreatment and the subsequent enzymatic saccharification treating single and mixed feedstocks [15, 18]. Besides the hydrolysis route using enzyme as catalyst following IL pretreatment, the conversion of biomass in ILs can be realized chemically through the acid catalysis [6, 19, 20]. It is an enzyme-free, wash-free one-pot process where monomeric sugars can be directly released from plant cellulose and hemicellulose within 2–3 h using direct injection of acid and water after IL pretreatment. The significant reduction of processing time and elimination of the washing and enzymatic hydrolysis steps would be beneficial for biorefineries in terms of increased productivity and significant cost reduction. Additionally, there is no need for IL separation or solid/liquid separation before acidolysis which may take up the significant operation time and cost for large-scale process [19].

Recently, a collaboration between three national labs: Sandia National Laboratories (SNL), Lawrence Berkeley National Laboratory (LBNL), and Idaho National Laboratory (INL) successfully demonstrated efficient sugar production using acid-assisted IL deconstruction at milliliter scale using a blend of MSW with corn stover. A range of MSW/CS blends were also evaluated for the feedstock cost using least cost formulation model and a ratio of 20:80 was identified that met the 2017 cost target of $80/ton and quality specifications (ash content less than 5%) for efficient biochemical conversion [6]. However, the data generated for this research along with other IL acidolysis data to date were obtained at the 10–100 mL level of operation, which cannot be directly translated to industrially relevant scales. Thus, liter-scale experiments are a necessary intermediate step between bench and pilot scale to identify operational parameters and potential problems associated with scale-up prior to pilot-scale and full-scale commercial operations. In particular, scale-up of the one-pot acid-assisted IL deconstruction involves challenges such as reactor compatibility, mixing at high solid loading, handling of acid and biomass at high temperatures, sugar recovery and separation, etc., which have not been effectively resolved during the development stages at bench scale.

As the continuation of the collaborative work, the current study aims to (1) evaluate and address the engineering and operation challenges to scale up IL acidolysis process; (2) investigate the response and scaling effects of MSW/CS blends for sugar conversion; (3) optimize the process by integrating with efficient and scalable product separation and recovery process; and (4) provide baseline parameters to facilitate the further pilot-scale operations.

## Results and discussion

### Reactor compatibility with IL and acid biomass system

One of the process scale-up challenges is the reactor material compatibility with the reactant. In this study, corrosion effects to the reactor caused by ILs with the chloride anion and biomass reactions under the acidic conditions were unknown [21, 22]. Thus, it was critical to evaluate such reactor performance and understand the potential risks of chemical corrosion, before directly utilizing the reactor for scale-up efforts. Coupon testing was conducted using Hastealloy C276 (bioreactor construction material) obtained from commercial source.

**Fig. 1:**
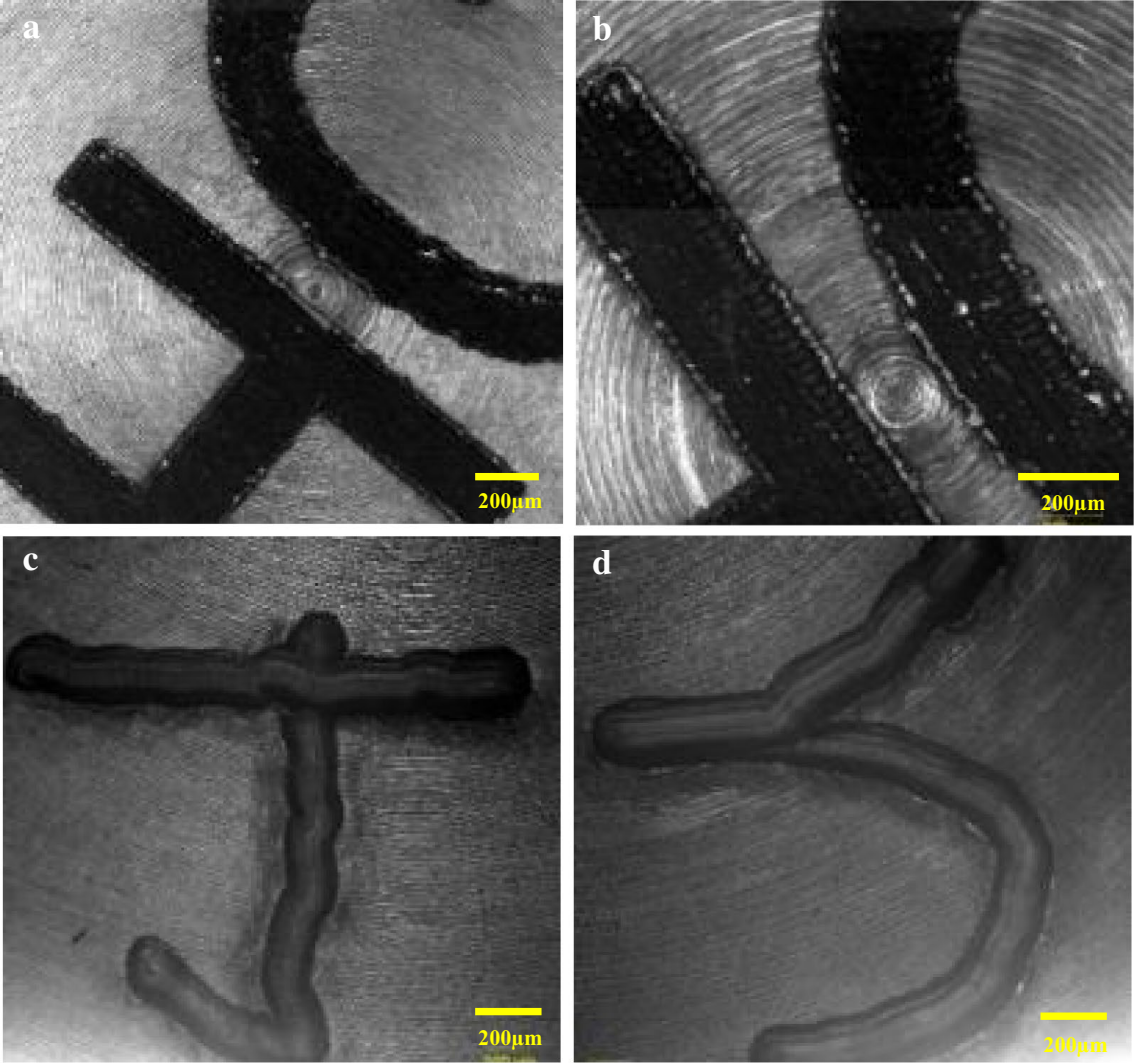
Microscopy images before and after 144-h IL acidolysis treatment showing the minor surface corrosion: **a** coupon surface before testing, **b** coupon surface after 24-h treatment in [C_4_C_1_Im]Cl, **c** coupon surface after 114-h treatment in [C_4_C_1_Im]Cl, and **d** coupon surface after 114-h treatment in [C_2_C_1_Im]Cl

The corrosion rates and weight loss for the two types of coupons (physical properties shown in Table 1) exposed to both [C_4_C_1_Im]Cl and [C_2_C_1_Im]Cl—biomass slurry for 144 h as a function of HCl concentrations are shown in Table 2 [21, 23]. The corrosion tests were performed in total six cycles of experiments to simulate the environment of biomass IL pretreatment at 140 °C and acidolysis at 105 °C. For acid concentration at 0.6%, the corrosion rates for all coupons are between 0 and 1 mile per year (mpy). The coupons in the [C_4_C_1_Im]Cl showed similar corrosion rates at 0.481 and 0.511 mpy while the [C_2_C_1_Im]Cl was less corrosive with a rate of 0.295 mpy. The weight loss and metal loss were very low, ranging from 0.013 to 0.048% and 0.005 to 0.008 mils, respectively. When acid concentration increased to 1.8%, the corresponding corrosion rates increased 2–3 times for all coupons. Similarly, [C_2_C_1_Im]Cl was less corrosive than [C_4_C_1_Im]Cl at higher acid concentration. Although weight loss and metal loss increased with the increasing acid concentration, the corrosion behavior was limited to an overall corrosion rate less than 1 mpy. Furthermore, microscopic examination of the coupon surface shows the morphologies of coupon surface (Fig. 1) after 144-h treatment in [C_4_C_1_Im]Cl/biomass slurry and 1.8% HCl. The images demonstrated that only very minor surface corrosion was observed. Long-term exposure of coupons in two ILs and 1.8% HCl was further performed for 4 weeks at room temperature, and little further corrosion was observed (Fig. 1). These results indicate while slight corrosion may happen during high-temperature reaction conditions, the overall corrosion impact under the current experimental conditions is not a severe concern for our Hastelloy C276 Parr reactor scale-up efforts.

**Table 1:** Physical properties of coupons used in corrosion testing

Physical properties				
Coupon types	Coupon source	Thickness (cm)	Surface area (cm^2^)	Density (g/cm^3^)
I	Parr	6.25	5.05	8.76
II	MacMaster	0.89	15.91	8.61

**Table 2:** Coupon testing during IL pretreatment (140 °C) and acidolysis (105 °C) under various acid concentrations and corrosion results

Reaction conditions	Test results					
Coupon type	Reaction time (h)	Ionic liquid type	HCl concentration (%)	Weight loss (%)	Metal loss (mils)	Corrosion rate (mpy)
I	144	[C_4_C_1_Im]Cl	0.6	0.013	0.008	0.481
II	144	[C_4_C_1_Im]Cl		0.048	0.008	0.511
II		[C_2_C_1_Im]Cl		0.029	0.005	0.295
I	144	[C_4_C_1_Im]Cl	1.8	0.028	0.017	1.055
II	144	[C_4_C_1_Im]Cl		0.073	0.012	0.770
II		[C_2_C_1_Im]Cl		0.066	0.011	0.670
I	366	[C_4_C_1_Im]Cl	1.8	0.001	0.0005	0.002
II	366	[C_4_C_1_Im]Cl		0.002	0.0004	0.006
II		[C_2_C_1_Im]Cl		0.003	0.0005	0.017

### Integrated one-pot IL acidolysis scale-up process

Imidazolium-based ILs have been shown great promise for dissolving and fractionating various types of feedstocks into fermentable sugars at small scales [6, 14, 16]. Chemical processing of feedstocks at larger scale involves the integration of feedstock feeding, mixing, processing, separation and recovery with pretreatment technologies. ABPDU has published studies that demonstrated the successful 600-fold scale-up of switchgrass, eucalyptus, and mixed feedstock IL pretreatment in a 10-L reactor with the process integration of pretreatment, water precipitation, homogenization, washing, centrifugation and the product recovery system which reduced the IL contents in the recovered biomass product and mitigated the risk to downstream enzymatic saccharification and microbial fermentation [15, 18]. These previous published works focused on the IL pretreatment followed by product recovery for enzymatic saccharification, which requires extensive washing to remove the residual IL and mitigate the inhibition to downstream processing. The excessive use of water and waste disposal associated with washing poses critical challenges for the scale-up of IL process.

**Fig. 2:**
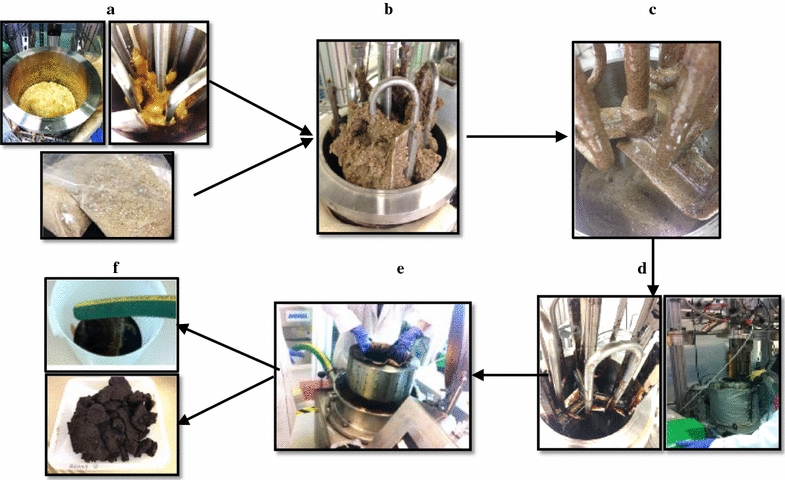
Process flow of IL acidolysis of MSW/corn stover blends at 6 L scale. Photographs depicting (**a**) IL preheating in Parr reactor and MSW/CS blend, (**b**) mixing of IL and biomass, (**c**) solubilization of blend in IL after 2 h at 140 °C, (**d**) acidolysis and incubation of MSW/CS blends, (**e**) basket centrifugation for solid/liquid separation, and (**f**) recovery of sugar hydrolysate and lignin-rich product

Building on the recent development of IL acidolysis which breaks down polysaccharides directly into pentose and hexose in the presence of IL [6, 19, 24], this work presented an improved scale-up process integration and realized the one-pot enzyme-free and wash-free sugar conversion process using mineral acid. Figure 2 presents images taken at different stages of the IL pretreatment, acidolysis and product recovery process. The MSW/CS (20:80) blend used in this study was previously evaluated for its potential to meet the cost target ($80/ton) and quality specifications (37.7% glucan, 18.6% xylan, and 4.6% ash) [6]. First, 3.06 kg of [C_4_C_1_Im]Cl or [C_2_C_1_Im]Cl was loaded into the 100-L Parr reactor and preheated to 70 °C for IL melting (Fig. 2a). Then, 0.34 kg (dry weight) of MSW/CS blends was loaded into the 10-L Parr reactor and mixed with IL at a solid loading of 10% (w/w) (Fig. 2b). The MSW/CS blend was observed to be significantly solubilized in IL after 2-h reaction at 140 °C as evidenced by the lack of biomass or MSW paper fibrous structure. This is similar to what was observed for the same reaction conditions in 10-mL small-scale reactions or 200-mL scale glass reactor [6, 19] (Fig. 2c). We attribute this finding to the effective and uniform mixing and heating capacity of the Parr reactor with the anchor impeller and internal temperature control. Figure 2d depicts the formation of sugar hydrolysate/IL mixtures with the injection of acid for direct hydrolysis in the reactor. The efficient recovery of the sugar-rich hydrolysate and removal of residual lignin-rich solid is a key step in the scale-up process and a basket centrifuge was used for solid/liquid separation (Fig. 2e). The centrifugation speed was set at 1200 rpm for 30 min to recover the sugar-rich hydrolysate and lignin-rich product (Fig. 2f). During each step, water was recycled by pumping back into the centrifuge and recirculating for 30 min at centrifugation speed of 250 rpm to obtain liquid washes and lignin-rich solids. In the end, centrifugation speed was increased to 1200 rpm for 30 min to recover the final solid product that was further analyzed for total weight and composition analysis. Overall, the successful integration of these unit operations, i.e., one-pot pretreatment and acidolysis under efficient mixing, liquid/solid separation, and material handling/washing, was essential to develop a baseline that will facilitate further development for a commercially scalable and cost-competitive process.

### Acidolysis of MSW/CS blends in [C_2_C_1_Im]Cl

**Fig. 3:**
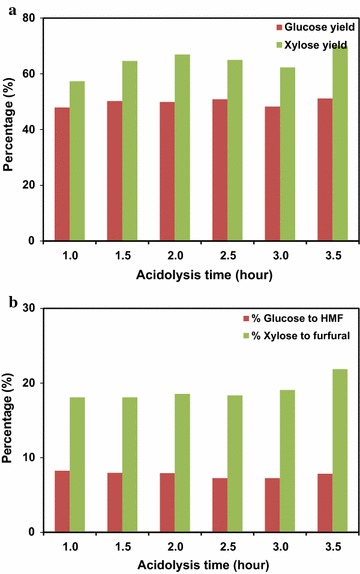
MSW/CS acidolysis with [C_2_C_1_Im]Cl pretreatment at 6 L scale. **a** Effect of acidolysis time on glucose and xylose yield, **b** effect of acidolysis time on sugar degradation

Scale-up of one-pot sugar conversion was first carried out using IL [C_2_C_1_Im]Cl for the acidolysis process. It is known that sugars can be lost due to spontaneous degradation to other small molecules during acidolysis. For example, glucose and xylose can be dehydrated to furans and other degradation products under acidic conditions [19]. To determine the extent to which this occurred during acidolysis, effect of acidolysis time (1–3.5 h) was evaluated to optimize the most efficient conversion time with the lowest levels of sugar degradation. The results are shown in Fig. 3a, b. The sugar yields obtained after pretreatment and acidolysis were calculated using Eq. (1):

1 $${\text{Yield}} \% = \frac{{C_{\text{sup }} \times M_{\text{sup }} }}{{W_{ } \times C_{ } \times f}} \times 100\%$$

where *C*_sup_ is the sugar concentration of the supernatant (w/w), *M*_sup_ is the mass of the supernatant, *W* is the weight of the biomass, *C* is the percentage of glucan or xylan contained in the biomass, and *f* is the factor to convert glucan or xylan to glucose or xylose (1.11 for glucan and 1.14 for xylan). For 3.5 h of acidolysis at 105 °C, the MSW blends converted in [C_2_C_1_Im]Cl achieved a glucose yield of 51% and a xylose yield of 71%. When increasing the acidolysis time from 1.0 to 2.0 h, both glucose and xylose yields slightly increased. Longer acidolysis time did not further improve the sugar conversion. 5-(Hydroxymethyl) furfural (HMF) and furfural were quantified in the supernatant for each acidolysis time. The results show that 8% of the glucose (equivalent) was converted to HMF after 1.0 h acidolysis, but more xylose (18%) was dehydrated to furfural. The sugar degradation was not sensitive to longer acidolysis times with no significant increase of HMF and furfural yields until the acidolysis time reached 3.5 h. Previous work at the 20-mL scale demonstrated that acidolysis of MSW/CS blend (25:75) resulted in a maximum 77% glucose and 51% xylose release [6]. It was also shown that more CS in the blend led to reduced sugar yields. In comparison to the small-scale study, the 6-L scale achieved lower glucose yields, possibly due to the higher CS ratio, and degradation of glucose. Xylose yield was higher since xylan is easier to dissolve and hydrolyze compared to glucan [6].

**Fig. 4:**
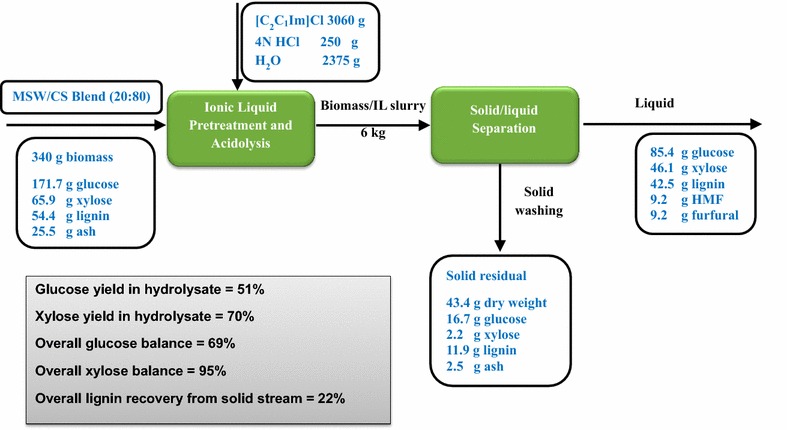
Mass balance of the MSW/CS blend acidolysis in [C_2_C_1_Im]Cl

A mass balance for the one-pot [C_2_C_1_Im]Cl pretreatment and acidolysis, the subsequent solid/liquid separation and product recovery through centrifugation, and their resultant composition of the products is summarized in Fig. 4 to provide a clear overview of this sugar production scale-up process. On the 340 g basis of untreated MSW/CS blend, the hydrolysate retained 85.4 g of glucose and 46.1 g of xylose with 9.2 g of each sugar degradation product, HMF and furfural, and 42.5 dissolved lignin in the IL/hydrolysate mixture. Furthermore, 43.4 g of solid residue was recovered after water washing, containing 11.9 g of lignin, a small quantity of ash (2.5 g), and undigested glucan (16.7 g in terms of glucose) and xylan (2.2 g in terms of xylose). The overall glucose yield on the basis of starting materials is 51%, lower than xylose (70%), which is attributed to the greater recalcitrance of glucan and easier depolymerization of xylan during the IL acidolysis. The overall glucose balance and xylose balance are 69 and 95% counting both solid and liquid fractions. During the pretreatment and acidolysis, a significant amount of lignin was also solubilized into the liquid stream, indicating potential opportunities for lignin valorization. For a long-term development of biorefinery, lignin supply will progressively increase as many types of lignocellulosic feedstock are implemented in the future. Adding value to the lignin utilization will significantly enhance the competitiveness of biomass-to-biofuel conversion [25–27].

### Acidolysis of MSW/CS blends in [C_4_C_1_Im]Cl

**Fig. 5:**
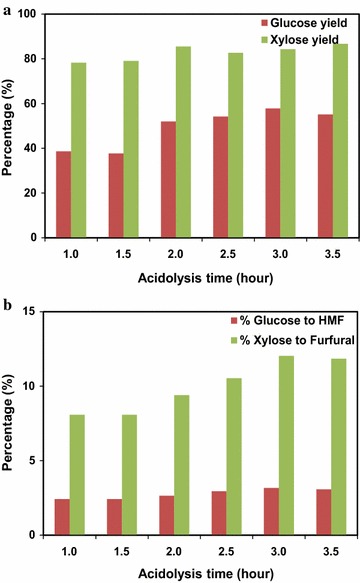
MSW/CS acidolysis with [C_4_C_1_Im]Cl pretreatment at 6 L scale. **a** Effect of acidolysis time on glucose and xylose yield, **b** effect of acidolysis time on sugar degradation

The acidolysis scale-up of MSW/CS blends was also conducted for the IL: [C_4_C_1_Im]Cl. Sugar yields and degradation to HMF and furfual were continuously monitored at various acidolysis time and the results are shown in Fig. 5a, b. It appeared that the [C_4_C_1_Im]Cl under the same pretreatment and acidolysis conditions (140 °C, 2 h and 10% solid loading; 100 mg HCl per g biomass, 105 °C, 3.5 h) has lower severity than [C_2_C_1_Im]Cl, as evidenced by the presence of small quantities of fibrous biomass, likely caused by the decreased accessibility of larger molecular IL to the plant cell wall for feedstock solubilization. However, the lower severity may help to reduce the sugar degradation during acidolysis step. As shown in Fig. 5a, higher glucose yield (58%) and xylose yield (87%) were obtained for [C_4_C_1_Im]Cl, in comparison to [C_2_C_1_Im]Cl under the same process conditions at 3.5 h of acidolysis. The glucose and xylose yields increased with the increase of acidolysis time from 1 to 3 h. Figure 5b further demonstrated less degradation of glucose to HMF (up to 3%) and xylose to furfural (up to 12%) during the 3.5-h acidolysis. Results also show that shortening acidolysis time helps to decrease the sugar degradation without sacrificing sugar production.

**Fig. 6:**
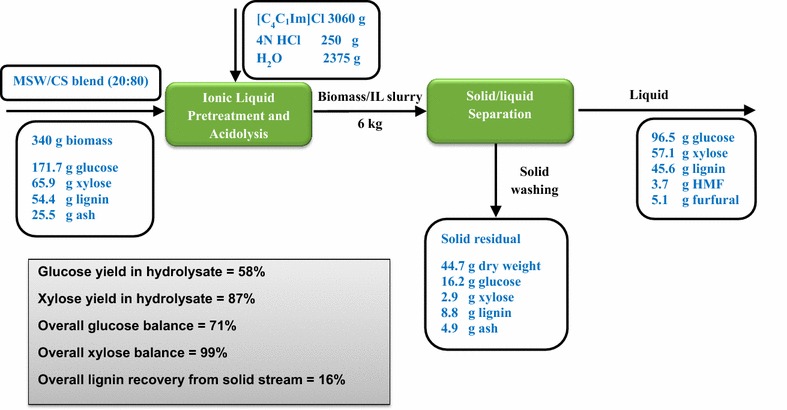
Mass balance of the MSW/CS blend acidolysis in [C_4_C_1_Im]Cl

Similarly, the mass balance for the [C_4_C_1_Im]Cl pretreatment and acidolysis, the subsequent unit operations including solid/liquid separation and product recovery, and their resultant composition of the products is summarized in Fig. 6. In comparison to the results of [C_2_C_1_Im]Cl, higher sugar contents were recovered in hydrolysate with 96.5 g of glucose and 57.1 g of xylose by [C_4_C_1_Im]Cl. Higher lignin solubilization (45.6 g) and less sugar degradation (3.7 g of HMF and 5.1 g of furfural) were observed in the hydrolysate. 44.7 g of solid residue contained similar amounts of undigested glucan and xylan, with lower lignin and higher ash. The overall glucose balance and xylose balance are 71 and 99% counting both solid and liquid fractions. The overall lignin recovery from solid stream is 16%, similar to [C_4_C_1_Im]Cl process, with the majority of lignin being dissolved in the liquid fraction for potential lignin valorization by IL-tolerant organisms or catalysts.

It should be addressed that due to their relatively high costs, recovery and recycle of ILs have been given more and more attention as a requirement for its commercial use in biomass deconstruction. The IL recovery approaches include using anti-solvents such as acetone, followed by distillation/evaporation for separation [28], biphasic system with addition of an aqueous solution containing kosmotropic anions, such as phosphate, carbonate, or sulfate [19], and sequential membrane filtration and vacuum evaporation post sugar extraction from aqueous IL hydrolysate [29, 30]. Although these results show that separation and recovery of IL can be achieved by various methods, to date, all these potential alternatives have been limited to the lab-scale level of development and require more investigation before scale-up can occur. The enzyme-free and wash-free one-pot sugar production process demonstrated in this paper does not involve IL recovery. However, these ILs are expensive and likely inhibitory to fermentation microbes so a recovery process will be required. Alternatively, Sandia, ABPDU/LBNL and INL are working together to use the lower cost biomass-derived renewable “bionic liquids” for conversion of these MSW blends into hydrocarbon fuels at both milliliter-scale and liter-scale studies. The present work provides an essential step to understand and evaluate the scale-up effects and important parameters that require further development toward a commercially scalable and cost-competitive process.

## Conclusions

MSW paper materials can be blended into CS providing lower cost and high-quality biorefinery feedstock inputs that are easily processed using the IL-based deconstruction technology. In this study, the acidolysis of MSW/CS blends into fermentable sugars with both ILs: [C_2_C_1_Im]Cl and [C_4_C_1_Im]Cl, was successfully scaled up by 30-fold at 6 L with a solid loading of 10%. The results are comparable with small-scale experiments that have been conducted previously and indicate that MSW blend feedstock is a viable and valuable resource to consider when assessing the biomass availability and affordability demands of the biorefineries scheduled for deployment. Reactor material was first evaluated for chemical compatibility and corrosion behavior in the chloride-rich acidic biomass/IL system and the results show that there are no fundamental issues in terms of performance associated with the scale-up of this MSW/CS blend to sugar conversion technology. An integrated scale-up process including one-pot pretreatment and acidolysis, product separation and recovery was effectively developed. Further process optimization at both lab and bench scale is desired to achieve high sugar conversion. The scale-up attempt and process integration will leverage the opportunity toward a cost-effective sugar/lignin production technology.

## Methods

### Raw materials

The paper waste materials, consisting of 15% glossy paper, 25% non-glossy paper, 31% non-glossy cardboard, and 28% glossy cardboard, were collected over the course of 2 weeks from an Idaho National Laboratory (INL) office building and utilized to represent the MSW paper material in this study. The material was shredded through a conventional office shredder and the cardboard material was cut into pieces with scissors. Each paper type was ground to 2 mm using a Thomas Scientific Model 4 Laboratory Wiley Mill (Thomas Scientific, Swedesboro, NJ). The corn stover was grown near Emmitsburgh (IA, USA) and was harvested in September 2010. Harvested corn stover was ground using a Vermeer BG480 grinder (Vermeer, IA, USA) designed for processing up to 4 × 4 ft bales. A 1-in. screen was used for all these grinds. The MSW paper materials were then mixed with ground corn stover in a ratio of 20:80, which was previously determined to meet the cost and quality targets [6]. The 1-Ethyl-3-methylimidazolium chloride, [C_2_C_1_Im]Cl, and 1-Butyl-3-methylidazolium chloride, [C_4_C_1_Im]Cl (>97% purity), and 6 N hydrochloric acid were purchased from Sigma-Aldrich.

### Coupon testing for chemical compatibility

Prior to utilizing the Parr reactors for ionic liquid acidolysis of biomass, a set of coupon testing experiments were conducted to evaluate the reactor compatibility and corrosion behavior of reactor Hastelloy C276 materials. One type of coupons was obtained from Parr Instrument Company (Moline, Illinois, USA) in the form of disk plates. Another type of coupon was purchased from McMaster-Carr Supply Company in the form of cylinders. Their sizes and physical properties are shown in Table 1. Two sets of coupon tests were carried out in two 50-ml global glass reactors (Syrris, UK), one for [C_2_C_1_Im]Cl and the other for [C_4_C_1_Im]Cl.

For each set of testing, in total six cycles of experiments were performed to simulate the environment of biomass acidolysis in IL. Each cycle, the biomass solutions were prepared by combining 3 g of biomass with 17 g IL, and coupons were placed into the mixture in duplicates. The reactor was programmed to heat up to 140 °C and hold for 2 h. The solutions were then cooled down to the acidolysis temperature of 105 °C and acidolysis started after 15 min equilibration time. Acidolysis was performed following a procedure described previously [19]. In summary, 2.07 mL 4 M HCl was added to the biomass/IL solution (*t* = 0) and with DI water added to give an H_2_O concentration of 5% (w/w) of the total weight. More water (3.175 mL) was added at 10 min to get the targeted water concentration of 20%. Water was injected into the mixture starting from 15 min at the rate of 227.5 μL/min for 45 min. Acidolysis was continued for a total of 2.5 h and stopped by cooling down the reactor to room temperature. After that the coupons were soaked in the reactor overnight to complete a 24-h cycle. After each cycle, the coupons were taken out, cleaned following the ASTM protocols [31], and measured the weight loss, and calculated the metal loss and the corrosion rate using the following equations:

2 $${\text{Corrosion}}\,{\text{rate}}\left( {\text{CR}} \right) = \frac{{{\text{Weight}}\,{\text{loss}}\,\left( {\text{g}} \right) *K}}{{{\text{Alloy}}\,{\text{density}}\,\left( {{{\text{g}} \mathord{\left/ {\vphantom {{\text{g}} {{\text{cm}}^{ 3} }}} \right. \kern-0pt} {{\text{cm}}^{ 3} }}} \right) * {\text{Exposed}}\,{\text{area}}\,\left( {\text{A}} \right) * {\text{Exposure time}}\,\left( {\text{h}} \right)}}$$

3 $${\text{Metal}}\,{\text{loss}}\, ( {\text{ML) = }}\frac{{{\text{Weight}}\,{\text{loss}}\,\left( {\text{g}} \right) *\,K}}{{{\text{Alloy}}\,{\text{density}}\,\left( {{{\text{g}} \mathord{\left/ {\vphantom {{\text{g}} {{\text{cm}}^{ 3} }}} \right. \kern-0pt} {{\text{cm}}^{ 3} }}} \right)\, *\,{\text{Exposed}}\,{\text{area}}\,\left( {\text{A}} \right)}}$$

where the *K* factor is 3.45 × 10^6^ for Eq. (2) and 393.7 for Eq. (3) [21, 31]. The coupons were placed back into the reactor to start another cycle of 24 h under the same acidolysis conditions. The six cycles were performed for total 144 h. Then, the coupons were continuously soaked in the IL/biomass solutions for 4 weeks to perform long-term corrosion test and measure weight loss and corrosion rate.

In addition, the coupons were also monitored at the end of each cycle for surface changes using a Zeiss LSM 710 confocal system mounted on a Zeiss inverted microscope (Carl Zeiss Microscopy, LLC, Thornwood, NY) with a 10× objective.

### Reactor modification, setup and operation for large-scale IL pretreatment

A 10-L Parr Floor Stand Reactor (Model 4558, Parr Instrument Company, Moline, IL, USA) made of Hastealloy C276 was used for this study. To realize the safe handling of biomass and acid/water injection at high temperature and induce the acidolysis in IL/biomass slurry, the Parr reactor was customized to accommodate the process scale-up challenges. First, the reactor was modified and installed with a self-sealing packed gland drive and anchor impeller to allow heavy duty mixing to facilitate the material uniformity at high solid loadings. Second, a sampling injection port was installed in the reactor to allow acid/water injection during the high-temperature operation, and safe material handling without opening up the reactor.

A 10% (w/w) biomass/IL solution was prepared by combining 340 g of biomass with 3060 g of IL in the Parr reactor. For each run, the reactor was sealed and the reactants were heated at 140 °C for 2 h with a stirring speed of 50 rpm from the anchor impeller. After 2-h incubation at 140 °C, the reactor was cooled to 105 °C with chilled water through the cooling coils inside the reactor. Temperature ramping (to 140 °C) and cooling (to 105 °C) times were approximately 30 and 15 min, respectively. Detailed procedure for IL pretreatment and acidolysis is summarized in Table 3. In brief, the acidolysis started after 15 min equilibration time, 4 N HCl was pumped into the biomass/IL solution through the injection portal and with DI water added to give a water concentration of 5% (w/w) of the total weight. More water was pumped and injected at 10 min to get the targeted water concentration of 20%. Water was injected into the mixture starting from 15 min for 45 min to get to the final water concentration of 43%. Then, the acidolysis was continued and incubated for another 2.5 h and stopped by cooling down the reactor to room temperature. Time points were taken every 30 min during acidolysis to monitor sugar yield, HMF and furfural production by HPLC.

**Table 3:** IL pretreatment and acidolysis conditions in 10-L Parr reactor

Pretreatment conditions	IL pretreatment process				Acidolysis process							
					0–10 min			10–15 min		15–60 min		60–210 min
	Solid loading (%)	Starting weight *W*0 (g)	Dry biomass (g)	IL (g)	HCl 4 N (g)	1st water addition to 5% (g)	Total weight *W*1 (g)	2nd water addition to 20% (g)	Total weight *W*2 (g)	3rd water pumping addition to 43% (g)	Total weight W3 (g)	Incubation at 105 °C
140 °C, 2 h	10%	3400	340	3060	250	0.0	3650	643	4293	1732	6025	Incubation

### MSW/CS blends composition and hydrolysate analysis

Moisture content of pretreated biomass was quantified using a moisture content analyzer (Mettler Toledo, Model HB43-S Halogen) by heating to 105 °C and monitoring the mass until it remained constant. Acid-insoluble lignin, and structural carbohydrates, i.e., glucan, xylan, arabinan and galactan, of MSW/CS blend (20:80) before and after IL pretreatment and acidolysis were determined according to analytical procedure of the National Renewable Energy Laboratory (NREL) by a two-step sulfuric acid hydrolysis [32, 33]. Briefly, 300 mg of biomass and 2 mL 72% H_2_SO_4_ were incubated at 30 °C while shaking at 300 rpm for 1 h. The solution was diluted to 4% H_2_SO_4_ with 56 mL of DI water and autoclaved for 1 h at 121 °C. The reaction was quenched by placing the samples into an ice bath before removing the biomass by filtration. Carbohydrate concentrations were determined from the filtrate by Agilent HPLC 1200 Series equipped with a Bio-Rad Aminex HPX-87H column and a Refractive Index detector, and acid-insoluble lignin was quantified gravimetrically from the solid biomass after heating overnight at 105 °C. Absorbance reading of acid-soluble lignin was taken using an UV–Vis spectrophotometer (Shimadzu UV-2401) with high-purity quartz cuvettes with a 1 cm pathlength [34].

Furfural and HMF in the hydrolysates was further analyzed using an Agilent 1200 High-Pressure Liquid Chromatography (HPLC) instrument equipped with Aminex HPX-87 H column and an UV detector (λ = 280 nm). Eluent containing 4 mM H_2_SO_4_ was used and the flow rate was 0.6 mL/min. Standard calibration curves were made by using six different known concentrations of furfural/HMF (125–1000 μM) from Sigma-Aldrich. Ionic liquid was quantified using reversed-phase liquid chromatography using an HPLC equipped with Eclipse Plus C8 column and Evaporative Light Scattering Detector (ELSD, evaporator temperature  =  45 °C, nebulizer temperature  =  30 °C; gas flow  =  1.2). All analyses were performed at 0.5 mL/min flow rate. The injection volume was 5 μL and the column temperature was 30 °C.

## Abbreviations

IL: ionic liquid
ABPDU: Advanced Biofuels Process Demonstration Unit
HPAEC: high-performance anion exchange chromatography
INL: Idaho National Laboratory
SNL: Sandia National Laboratories
LBNL: Lawrence Berkeley National Laboratory
NREL: National Renewable Energy Laboratory
